# Comparative Developmental Expression Profiling of Two *C. elegans* Isolates

**DOI:** 10.1371/journal.pone.0004055

**Published:** 2008-12-31

**Authors:** Emily J. Capra, Sonja M. Skrovanek, Leonid Kruglyak

**Affiliations:** 1 Lewis-Sigler Institute for Integrative Genomics, Princeton University, Princeton, New Jersey, United States of America; 2 Department of Molecular Biology, Princeton University, Princeton, New Jersey, United States of America; 3 Howard Hughes Medical Institute, Princeton University, Princeton, New Jersey, United States of America; 4 Department of Ecology and Evolutionary Biology, Princeton University, Princeton, New Jersey, United States of America; National Cancer Institute at Frederick, United States of America

## Abstract

Gene expression is known to change during development and to vary among genetically diverse strains. Previous studies of temporal patterns of gene expression during *C. elegans* development were incomplete, and little is known about how these patterns change as a function of genetic background. We used microarrays that comprehensively cover known and predicted worm genes to compare the landscape of genetic variation over developmental time between two isolates of *C. elegans*. We show that most genes vary in expression during development from egg to young adult, many genes vary in expression between the two isolates, and a subset of these genes exhibit isolate-specific changes during some developmental stages. This subset is strongly enriched for genes with roles in innate immunity. We identify several novel motifs that appear to play a role in regulating gene expression during development, and we propose functional annotations for many previously unannotated genes. These results improve our understanding of gene expression and function during worm development and lay the foundation for linkage studies of the genetic basis of developmental variation in gene expression in this important model organism.

## Introduction

Microarray analysis of gene expression has provided considerable insights into biological processes. The data generated through this method allows predictions of function to be made for previously uncharacterized genes based on the observation that genes with similar function often show a common expression pattern. Thus, genes of unknown function that are expressed similarly to a set of genes whose function is known can be putatively assigned to a gene ontology (GO) term [1], [2]. Ten years after the *C. elegans* genome was sequenced [3], it remains underannotated when compared to several other model organisms, both in number and quality of annotations [4]. Genes whose functions remain unannotated in the *C. elegans* genome likely have no obvious phenotypes when singly deleted, as there have been several genome-wide RNAi screens for function [5]–[7].

Although *C. elegans* development has been studied extensively, many of the studies were done in the pre-genomic era. In the past ten years, several groups have completed microarray time courses examining various aspects of *C. elegans* development [8]–[14]. However, many of the studies used PCR products to create arrays that only cover a fraction of the genome and assayed worms grown in liquid culture, which can affect expression [10], [12]–[14]. In comparison to the *Drosophila* data that showed nearly every gene changing over developmental time, the early studies identified relatively few genes that showed significant variation based on stage [15]. The more recent studies have used specific deletion strains to test for downstream components of known signaling pathways thus also identifying a relatively small number of developmentally regulated genes [8], [9].

Additionally, in using microarray data from a time course, it is important to recognize the temporal nature of the data. Most time series data is currently analyzed using methods that were initially developed for static data, and while these methods have provided biologically meaningful insights, they assume that each of the data points is independent [16]–[19]. Methods such as K-means clustering require *a priori* knowledge of the number of clusters into which the data should be divided.

In this study we compared transcript levels across development between two genetically divergent isolates of *C. elegans*, N2 (Bristol) and CB4856 (Hawaii) [20], [21]. These isolates differ at approximately one polymorphism per kb, a degree of genetic variation mirroring that found in the human population, both in amount and type [22]–[27]. We used high-quality oligonucleotide arrays that comprehensively cover known and predicted *C. elegans* genes, and applied methods specifically developed to analyze time courses and to discover regulatory motifs in order to identify developmentally regulated genes and to divide the data into biologically meaningful clusters. By studying a wild isolate in addition to the laboratory strain we are able to identify the types of genes that vary over development and to assess the amount of natural variation in expression levels present in the *C. elegans* population.

## Results

### Nearly all *C. elegans* genes show differential expression over development

**Figure 1:**
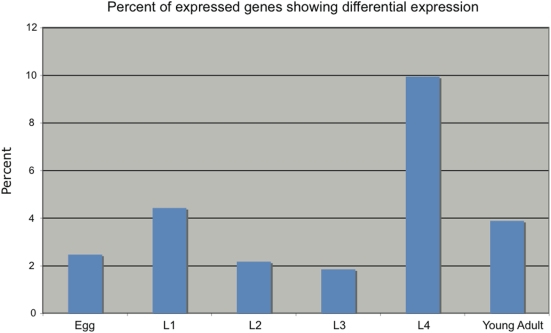
The L4 stage displays the largest variation in expression levels between strains. Each stage was analyzed separately. The dataset was filtered for genes that are present in all of the arrays for each stage. The number of expressed genes is different for each time point. A one-way ANOVA was used to identify genes that varied by strain; p-values of less than .01 were called significant.

In order to investigate *C. elegans* development on a genome-wide scale we used Agilent 4×44k *C. elegans* oligo microarrays to measure expression of 13,474 genes at 6 different developmental time points (egg, L1, L2, L3, L4, and young adult) in two different strains (N2 and CB4856). We analyzed the data using a two-way ANOVA to identify the relative impact of strain, stage, and strain by stage interaction on the observed transcript levels. 12,390 (91.9%) transcripts were found to have a significant stage effect, 2,797 (20.8%) were found to have a significant strain effect, and 283 (2.1%) were found to have a significant strain by stage interaction term (q<.05, Table S1). A strain by stage interaction term indicates that the developmental pattern of expression for that gene differs between N2 and CB4856. To determine which stage has the largest number of transcripts whose expression levels differ between N2 and CB4856 we analyzed each stage separately. A one-way ANOVA was used to measure the contribution of strain towards the observed variation. As can be seen in Figure 1, variation in transcript levels between N2 and CB4856 is the highest during the L4 stage, a finding consistent with the idea that selection is relaxed during the later developmental stages [28]. Overall, 2,211 genes are expressed differently between N2 and CB4856 in at least one stage. Most of the strain effect is not due to large-scale deletions of the gene in question, as only 88 genes in the dataset were identified as deleted in CB4856 [27].

### Much of the observed variation due to strain by stage effects is due to differential expression of innate immunity genes

**Figure 2:**
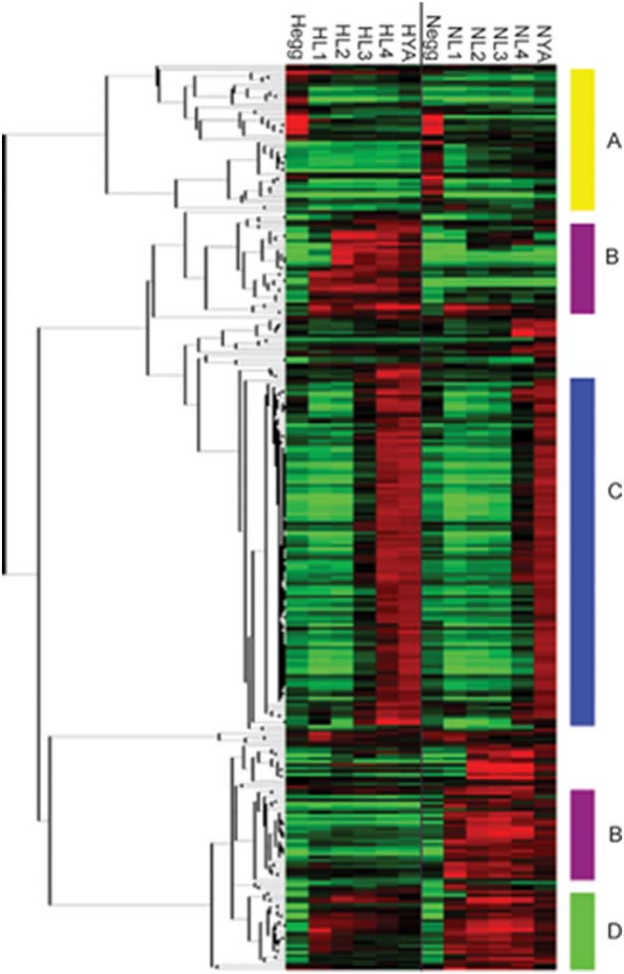
Genes that display significant strain by stage variation fall into four main categories. The genes that show significant variation due to strain by stage interaction were clustered hierarchically. Four distinct patterns appear in the clustered data, identified by the letters A–D. CB4856 (H) are on the left, from the egg to the young adult, while N2 (N) are on the right, from the egg to the young adult. Missing values were imputed using KNN-impute and expression values represent the average from four replicates.

Four general expression patterns emerged when genes displaying a strain by stage interaction were clustered hierarchically (Figure 2, Table S2). One set contains genes that are mostly expressed only during the egg stage of either N2 or CB4856 (A). Two sets of genes (B) are mostly only expressed in either N2 or CB4856. One set of genes turn on roughly one stage earlier in CB4856 than in N2 (C), while another set of genes turn off earlier in CB4856 than in N2 (D).

Several gene families are enriched in this set of genes, including: Math-BTB (p = .002), Duf-19 (p = 2.4e-5), major sperm proteins (p = .0035), protein tyrosine kinases (p = .000953), protein tyrosine phosphatases (p = 6.21e-15) and serine/threonine kinases (p = 4.03e-7). Genes containing Math-BTB or DUF-19 domains are preferentially deleted in CB4856 when compared to the N2 genome, relative to other gene families [27]. The differentially expressed protein tyrosine kinases and phosphatases are members of cluster C (Figure 2) and are expressed earlier in CB4856 than in N2, as are the major sperm proteins.

Many of these gene families have been implicated in innate immunity [29], [30]. Members of the C-type lectin and the pathogenesis related protein families are also present in the genes that display strain by stage variation. In order to test whether genes that have been implicated in the innate immune response are significantly enriched in this set, a list of genes that are upregulated in response to pathogen exposure was curated by hand, mainly from recent microarrays investigating the response of N2 young adults to multiple types of pathogens [Table S6, 29], [31], [32]. The genes showing a strain by stage interaction display significant enrichment of genes that have been implicated in innate immunity (p = .00215).

**Figure 3:**
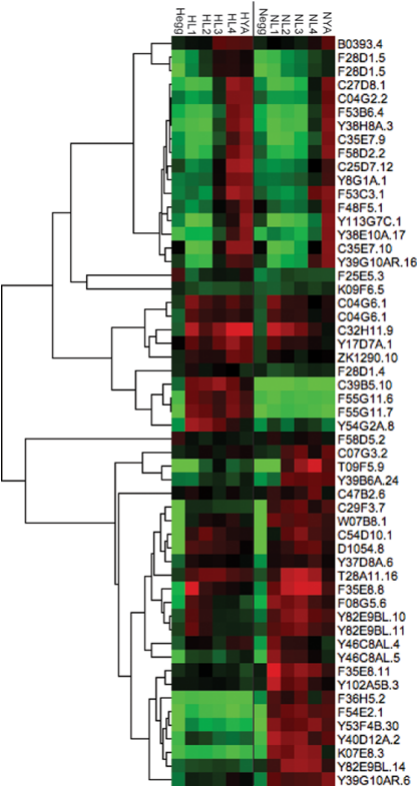
Innate immune response genes show several patterns of expression. Genes showing significant strain by stage variation that were directly implicated in the innate immune response were clustered hierarchically using a centered Pearson correlation and centroid linkage. CB4856 (H) are on the left, from the egg to the young adult, while N2 (N) are on the right, from the egg to the young adult. Each data point is the average of four biological replicates.

Of these genes, none are highly expressed during the egg stage (Figure 3). Every other pattern observed in the strain by stage significant genes is seen in this subset, including a set of genes that are highly expressed in one strain, but expressed at a very low level in the other. This could be indicative of the different pathogens that each strain is exposed to in nature, or due to different mechanisms that each strain uses in response to the same pathogen.

### Clustering identifies temporal patterns enriched for GO-terms associated with development

**Figure 4:**
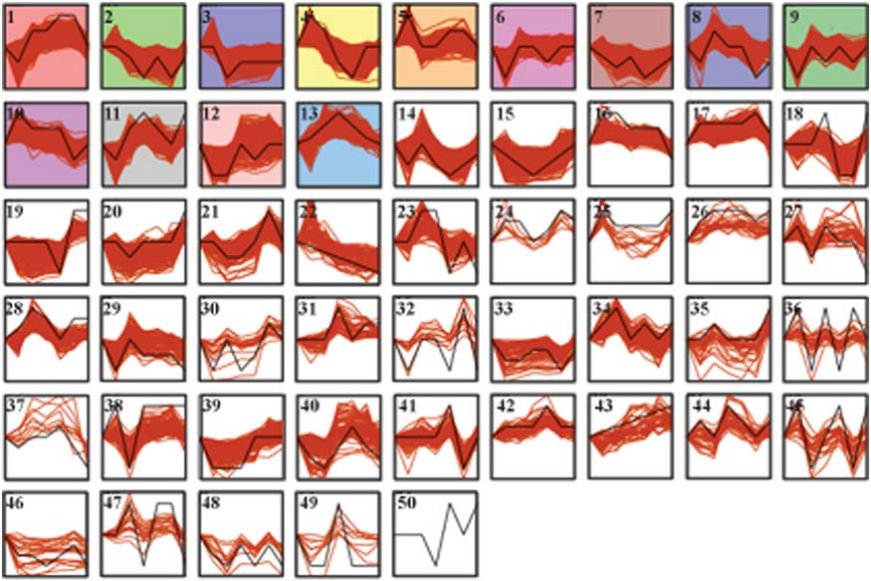
Thirteen significant expression profiles are found in the N2 data. The N2 expression data was loaded into STEM, which used 50 model profiles to cluster the data. The profiles are in order of statistical enrichment for genes matching the model profile. The model profile is in black while the gene expression patterns for each gene within the cluster are in red. The colored profiles are statistically significant, with p-values ranging from greater than 1e-226 to 7e-10. The number of genes assigned to each significant cluster ranges from 2,037 to 303. 9,593 genes were placed into a significant cluster. Clusters with similar colors show similar patterns. All expression profiles have a zero time point added to them so as not to force the egg stage to be the zero time point. The clusters were numbered prior to placing genes into clusters, and thus the numbers of the clusters are not correlated with significance.

The Short Time series Expression Miner (STEM) was used to cluster the microarray data due to its ability to take into account the temporal nature that is inherent in a developmental time course [33]. STEM selects a set of model profiles independent of the data, and the algorithm decides which model profile best fits the expression pattern for each gene. The genes are distributed among the clusters without regard to the number of model profiles used, and the algorithm takes into account that the time points are ordered and are not independent measurements. For the rest of the analysis, only the N2 data was used in order to allow for the comparison of these results to previous data and to avoid the complications of having strain effects obscure stage effects. Over 9,000 genes cluster into significant STEM clusters. When the CB4856 data is used, the resulting clusters are similar to those obtained using the N2 data, as the expression of most transcripts mainly vary by stage (data not shown). 13 of the 50 model profiles have a larger than expected number of genes assigned to the cluster and are called significant (Figure 4).

The clusters recapitulate known biology. Clusters 2–5 and 7 show significant enrichment for genes that are in the gene ontology (GO) category of multicellular organismal development, which is the most significantly enriched GO-term, as well as embryonic development ending in birth or hatching. Clusters 2 and 4 are also enriched for the GO-terms larval development (sensu nematoda) and post-embryonic development. The general developmental role can be broken down further. Cluster 2 shows enrichment for genes related to hermaphrodite genitalia development and sex differentiation. This cluster shows a peak in the egg stage as well as the L3 and young adult stages. It is known that the cells involved in the development of the gonad are born in the embryo, differentiate during L3 and have matured by the time egg laying begins in the young adult stage [34].

Combining current knowledge with the clustering results can also help to uncover potentially novel biology. Clusters 3, 4 and 7 are all enriched for cell-cycle response genes and for terms related to development. However, their different profiles may indicate different functions within the larger class of genes relating to the differential timing of developmental programs. Cluster 7 is the most complex of the three clusters, with many GO-terms associated with the genes in the cluster, ranging from M phase to intracellular organelle part. The genes belonging to cluster 3 appear to be mostly responsible for the early embryonic cell divisions and patterning as they are also enriched for P-granule function and pole plasm location, both of which are extremely important in *C. elegans* early lineage specification [35]. Cluster 4 genes are also expressed mainly in the egg stage; however, their functions appear to be broader, related to differentiation of cells into organs and tissues. In addition to cell-cycle related GO-terms, the terms cellular developmental part, organ development, morphogenesis of an epithelium, anatomical structure regulation, and cell differentiation are all enriched. Genitalia development and regulation of vulval development are also enriched, perhaps suggesting slightly different function for these genes during expression in the L4 and young adult stage. By breaking down a single biological function, the cell cycle, into different sets of genes through clustering, it becomes possible to dissect broader functions into narrower ones.

One surprising observation from this data is the prevalence of GO-terms related to neuronal development or function that appear in the developmentally organized clusters, especially clusters whose expression peaks after the embryonic stage. 80 new neurons are born during the larval stages of development—mainly during the L1 and the L2 larval stages, although the PDA neurons are born during L3 and several neurons that are born in the embryonic stage have been shown to reverse polarity in the larval stages [34]. Cluster 8 contains 501 genes whose role appears to be in neuronal activity. Broad aspects of neuronal activity are represented in this cluster. Terms from extra-cellular glutamate-gated ion channel to neurological system process to ion transport to axon function are all enriched. This set of genes is interesting because it is also enriched for terms such as pharyngeal pumping and eating behavior. UNC-73, a neurotransmitter that has been shown to be required for the regulation of pharynx pumping, is a member of this cluster [36]. Several of the eat genes [37] are also found in this cluster. The genes in this cluster are expected to be important for neural function, rather than creation, as no new pharyngeal neurons are created after hatching [34]. Expression of this cluster of genes is highest in the L1 stage, when the worms begin feeding. Lower expression in subsequent stages is presumably due to the creation of stable proteins. Cluster 13, which shows peak expression level during the L3 stage, is also enriched for GO-terms related to neural function, including neurotransmitter activity and ion channel activity. Rather than functioning in neural creation, this cluster of genes appears to be enriched for signal transduction of neuronal responses. GO enrichments for cluster 13 include neurotransmitter receptor activity, postsynaptic membrane and transcription factor activity. Thus these genes appear to act in response to neural signals and perhaps help to create, or maintain, synaptic connections. A complete list of significant GO-terms for all of the significant clusters can be found in Table S3.

### Genes in clusters contain motifs with a known developmental function, as well as novel motifs

In order to identify motifs important for developmental regulation of expression levels, the genes belonging to the significant STEM clusters were analyzed using FIRE [38]. FIRE uses mutual information to look for enrichment of motifs in both the 5′ promoter region and the 3′ untranslated region (UTR) in one cluster compared to the rest of the clusters. Motifs found in the 5′ region are expected to be binding sites for transcription factors or other DNA binding proteins that affect transcription, while motifs in the 3′ UTR are expected to be sites for regulation of mRNA by microRNAs or RNA binding proteins. The FIRE analysis was implemented using the STEM clusters described above (Figure 4), as well as with clusters that are enriched for GO-terms related to development and computed without the young adult time point (clusters not shown). This was done to ensure that the young adult time point did not drive the clustering.

59 motifs were found in the complete dataset—14 motifs from the 3′ UTR and 45 motifs from the 5′ end. Of these motifs, nearly half are highly conserved (conservation index >.9) between *C. elegans* and *C. briggsae*. The conservation score measures the fractions of 7-mers that are better conserved than the motif, i.e. a conservation score of 1 means that the motif is better conserved than all 7-mers. For the data without the young adult stage and using only the significant clusters from STEM that were enriched for GO-terms pertaining to development, 19 motifs were found—11 from the 5′ promoter region and 8 from the 3′ UTR. Of the 19 motifs found in the smaller dataset, 7 were also found in the larger one. Some of the motifs that were seen in the smaller dataset, but not the larger, could have been obscured through the inclusion of many more genes not directly related to development. As much of worm biology is still unknown, the exclusion of clusters not directly relating to development could also obscure relevant biology. This is shown by the fact that several known developmental motifs are found in the larger dataset but not in the smaller.

The FIRE analysis is able to recapitulate known biology. For example, the HNF-4 motif was found in both analyses. HNF-4 is the hepatocyte nuclear factor 4, a nuclear hormone receptor that is the vertebrate homolog to the *C. elegans* NR2A4 family [39], [40]. Nuclear hormone receptors (NHR) are important for a broad range of developmental phenotypes in multiple species [41]–[43]. The *C. elegans* NHRs that are most homologous to the vertebrate HNF4 are classified as belonging to the supplementary nuclear receptor (supnrs) group I, which consists of 24 members [40]. All members of this class share a conserved DNA binding domain and thus may bind the same stretch of DNA while interacting with other proteins to lead to differential patterns of expression both temporally and spatially [44]. NHR-40, which is a member of this family, is required for the development of muscle cells [44]. NHR-60 appears to be a maternally deposited mRNA and is required for embryogenesis and early larval development [45]. Many supnrs are expressed in the gut, possibly demonstrating a conserved function in intestinal development [46]. NHR-64 and NHR-69, which show the closest homology to vertebrate HNF-4, have no obvious phenotype in RNAi knockdown experiments, but NHR-64 is expressed in the neurons while NHR-69 is expressed mostly in the gut [47].

The E2F motif was found in the dataset that did not include the young adult time point. E2F proteins have been shown to be important for cell-cycle progression and development in mammals [48]–[50]. Kirienko and Fay [8] identified this motif as enriched in LIN-35 responsive genes, many of which involve cell-cycle regulated genes. E2F has also been shown to promote programmed cell death, which is important in the maturation of larvae [51] and is required for embryonic asymmetry [52]. The predicted targets of E2F are enriched for the GO-terms development and embryonic development.

The GATA-1 motif found in the larger data set belongs to the GATA transcription factor ELT-2 that is known to regulate most intestinal development in *C. elegans* [32], [53]. The GATA-1 motif was also observed in LIN-35 regulated genes that do not contain an E2F motif, possibly due to the role of LIN-35 in pharyngeal development [8], [54]. Other previously identified transcription factors identified in this analysis include EVI-1 and ADR-1. The EVI-1 homolog in *C. elegans*, EGL-43, is necessary for the AC/VU cell fate specification [55], while ADR-1 is highly expressed in the developing nervous system and vulva [56].

MicroRNAs (miRNA) are known to play a large role in *C. elegans* and vertebrate development [57]. 31 motifs were found in the 3′ UTR, of which two correspond to previously identified miRNAs. While the functions of the miRNAs identified through FIRE are currently unknown, the miRNA field is just maturing and there is still much that is not known about the roles of various miRNAs. miR-43 displays a stage dependent expression pattern; it is most highly expressed during the egg stage and not expressed at the adult stage [58]. miR-238 and 239 show the opposite expression pattern, in that they are expressed at a low level in the egg stage and at higher levels during subsequent stages [58]. miR-238 and 239 targets show enrichment for neurotransmitter binding, signal transducer activity, and localization to the membrane. It is possible that these miRNAs are partially responsible for neuronal development or synaptic maintenance.

In addition to the miRNA binding sites, several other motifs were identified in the 3′ UTR. There are over 500 RNA-binding proteins that have been annotated in the *C. elegans* genomes, most of whose targets and binding sites remain unknown, and many of which have developmental phenotypes when deleted [59]–[63]. A complete list of GO-term enrichments for the putative targets associated with the predicted motifs can be found in Table S4.

Of the motifs identified by FIRE, there are seven new 5′ motifs and two novel 3′UTR motifs found in sets of genes that are significantly enriched for terms relating to development, including: positive regulation of growth, development, embryonic development, development (sensu metazoa), organ development, reproduction, hermaphrodite genitalia formation, cuticle components, and signaling and ion channels (Tables 1, 2, 3, S4). Additional new motifs show functions that are probably not related to development, including nucleotide binding, ATP binding and protein modification (Table S4). One example of a novel motif is [ACT]CAC[AT]C[AC][CT]A which is enriched in clusters 17 and 41. Like the clusters, the targets of these motifs are enriched for GO-terms such a synapse part and neurotransmitter activity.

**Table 1:** Motifs identified by FIRE in the promoter region.

Motif	Location	Position Bias	Orientation	Motif name/ development
.[ACT]A[CT]GCGC	5′	Y		Reproduction
[AG]A[AGT]CGC[AT][CG].	5′	Y		
.[AG]ATCGAT[AT]	5′			CDP
[ACT][CT]GCG[GT][AC]C	5′	Y		
[CGT]CG[AC]GA[AT][CG][AGT]	5′	Y		HSF
[ACT][GT][CT]AACGA.	5′			
.AACA[ACT]CG.	5′		←	
[AG]A[AGT]ATCGG[CT]	5′		←	
.C[CT]TATCA.	5′	Y		GATA-1
[CT]AG[GT][CT]AG[AGT][CGT]	5′			Cuticle component
.TTTCAAA.	5′	Y		Ion channel activity
.A[CG][AT]TAAG[ACT]	5′			EVI-1
[AT]AAG[AGT]TCA.	5′			HNF-4
[ACT]A[CG]TA[CGT]AC[ACT]	5′			
[ACT]TT[GT]ATAC.	5′	Y	←	TATA
[CGT][ACG]ACC[CT]A[CG][ACT]	5′			RORalpha2
[ACG][CT]CT[AT]A[GT]A.	5′			
[AT]C.TAT[ACG]C[AT]	5′			Cuticle component
[ACT][CT]AAC[AC]C[ACT]T	5′			
.TTTTGAC[ACT]	5′		→	
[ACT]ACCAAAA.	5′		→	
.AC[AG]TCAT.	5′	Y	→	v-Jun
.C[CT][AC]CCC[CT][ACT]	5′			RREB-1
[AGT]CG[ACG]GAG[AG][ACG]	5′			
TCCACG[ACG]	5′			O2
.CGA[AC]G[AC]A[ACT]	5′	Y		Reproduction
.C[ACG]G[AG]ATC[CGT]	5′	Y		
.A.CGGAG[ACT]	5′			
[AT]CG[AG]A[AGT]TA[ACT]	5′			
.AT[AC]GAGC.	5′		→	
[CT].A[GT]CTAC[ACT]	5′		→	
.G[GT]ACTCC[AT]	5′			ADR1
ACT]GTGCGCC[ACT]	5′			
[CGT][ACG]A[CT]AGTA[AGT]	5′			
.TTGCCA[AC][ACT]	5′			
.TCAT[AT]AC.	5′		→	Cuticle component
.ATA[GT]C[AC][CG][CT]	5′		←	
[ACG][AG]CTTA[GT]A.	5′			
[ACT]CAC[AT]C[AC][CT]A	5′			Neurotransmitter
.[CT]ATGTAA.	5′		→	EFB4
[AGT][AT]CAG[AT]CT[CGT]	5′			
.CCTGAAA.	5′			
.CCGTAA[AG][CGT]	5′		←	
.C[ACT]CTAG[CT][ACT]	5′		→	
[AT]CTTAG[CGT][AC][AG]	5′			

Clusters from STEM clustering of all of the N2 data were used as FIRE input. 45 motifs were found in the promoter region. Named motifs are listed; motifs with targets that had significant GO-term enrichments for terms related to development are also listed.

**Table 2:** Motifs identified by FIRE in the 3′ UTR.

Motif	Location	Position Bias	Orientation	Motif name/ development
.[ACT][GT].CCCC.	3′ UTR		→	
.A[AC]ATAA[CT][AGT]	3′ UTR		→	Ion transport
[ACT]T[AGT]CCTCT.	3′ UTR		→	
[AGT]A[AC]T[AG][AT]GA[ACT]	3′ UTR		→	miR-43/ miR-250
.TC[CT]CAAC[ACT]	3′ UTR		→	
.CTCA.[AGT]T[CT]	3′ UTR		→	
[CGT]CG[ACT][GT]T[ACT][AT]C	3′UTR		→	
[GT][GT]TA[AT][AC][GT][AC][GT]	3′ UTR		→	
.T[CT][CG][AT][CT]GT[GT]	3′ UTR		→	Positive regulation of growth
[AC]ATC[GT]CT[AT][CT]	3′ UTR		→	
.A[AG][CT]AA[AG][CG].	3′ UTR	Y	←	
.AA[AC]A[AC][CG]T[AGT]	3′ UTR		→	
.A[CG][ACG]TA[CGT][AG][ACT]	3′ UTR		→	
[AG]A[CT]A[AG]AT[CT].	3′ UTR	Y	→	

Significant STEM clusters from all of the N2 data were used as FIRE input. 14 motifs were found in the 3′ UTR. Named motifs are listed, as are motifs whose targets show enrichment for GO-terms related to development.

**Table 3:** Motifs identified by FIRE using clusters without young adult data.

Motif	Location	Position Bias	Orientation	Motif name/development
..[AU][CU]CCCC.	3′ UTR		→	Development
.[AG]ATCGAT[AT]	5′			CDP
[CGT]CG[AC]G[AGT]C[CG][CGT]	5′			Organ development
.[AC][ACT]CGCTC.	5′			BSAP
.TTC[CG]C[AG]C[ACG]	5′			E2F
.CTGAAAA.	5′		→	Embryonic development
[ACT][ACG]CGTGA[AC].	5′	Y	→	Pax-6
.A[AC]UAA[AG]U.	3′ UTR	Y	→	
.GA[AC][CU][AGT]G[AC][ACG]	3′ UTR	Y	→	
.[ACU]GA[AG]G[ACU][GU][ACG]	3′ UTR		→	
[ACG]A[AGT]ACCA[AC]	5′		←	
[CT]C[CT][AC][ACT]CCC[ACT]	5′		→	
.ATA[CG]ATA[CGT]	5′		←	NIT2
.[AC]U[AG][AG][AG]CA.	3′ UTR		→	
[ACG]UA[AU][CGU]UAU.	3′ UTR		→	
.ATCAAAA.	5′		→	Cuticle component
[ACU][AG]UCC[CU]A[GU][ACU]	3′ UTR		→	
.AACTTTG.	5′	Y	→	HNF4
.A[AG][AU]AC[AC]A.	3′ UTR		→	miR-238/ miR-239

N2 expression data from the egg to the L4 stage was clustered using STEM. The clusters that showed enrichment for a GO-term associated with development were then used as input for FIRE. FIRE found 19 motifs in the data, 11 motifs in the 5′ region and 8 motifs in the 3′ UTR. Named motifs are listed, as are motifs whose targets show enrichment for GO-terms related to development.

### New GO annotations can be proposed for many genes

When compared to the yeast genome, the worm genome is severely underannotated with regard to gene ontology. Of the roughly 9,000 genes that are placed into significant clusters by the STEM algorithm, over 2,600 genes currently have no GO-annotations of any kind in the most current release of the ontology (4/20/08). Using clustering and motif analysis to divide genes into sets of putatively functionally related genes, and applying prior knowledge regarding the function of the known motifs, it becomes possible to propose annotations for previously unannotated genes. The motif analysis is useful because it can be used to divide clusters into smaller classes with more narrow functions. We propose general annotations for these 1,568 previously unannotated genes (Table S5).

For example, cluster 7 is composed of genes responsible for reproduction and development, but also genes responsible for mismatch repair and other cell-cycle functions that require DNA binding. While both functions are closely related, they represent distinct cellular processes. By using motif analysis, we are able to separate the functions in genes with no known function.

We propose that those genes in cluster 7 with the motif [AT]CG[AG]A[AGT]TA[ACT] are involved in nucleotide binding, while those with the motifs [CGT]CG[AC]GA[AT][CG][AGT], .[AG]ATCGAT[AT], .[ACT]A[CT]GCGC and .CGA[AC]G[AC]A[ACT] are involved in processes that are related to reproduction (Table 4). It is possible to further narrow the putative functions of unannotated genes. Along with the GO-terms related to reproduction, genes with the motif .[ACT]A[CT]GCGC are also highly enriched for terms related to organ development (Table S4). As cluster 12 as a whole is highly enriched for organ development, including hermaphrodite genitalia development, we propose that the genes in cluster 7 with this motif likely function in organ development. In addition, the motif .[AG]ATCGAT[AT] is the binding site for the CDP/Cut-like transcription factors. *Ceh-44*, which is the *C. elegans* homolog, is known to function in neuronal development and is enriched in larval neurons and thus the genes that possess this motif in their promoter regions are likely involved in neuronal development [64], [65]. Finally, cluster 7 is also enriched for GO-terms involved in meiosis and gamete generation, as are the genes with the motif [CGT]CG[AC]GA[AT][CG][AGT].

**Table 4:** Best motifs for a subset of genes in cluster 7.

Name	Probable function	Motif most likely to be functional
AC8.1	gamete generation	[CGT]CG[AC]GA[AT][CG][AGT]
B0001.5	gamete generation	[CGT]CG[AC]GA[AT][CG][AGT]
B0393.3	organ development	.[ACT]A[CT]GCGC.
C01A2.6	reproduction	.CGA[AC]G[AC]A[ACT]
C01G5.2	reproduction	.[ACT]A[CT]GCGC.
C04G6.4	gamete generation	[CGT]CG[AC]GA[AT][CG][AGT]
C06A5.6	reproduction	.CGA[AC]G[AC]A[ACT]
C07A9.7		[AG]A[AGT]ATCGG[CT]
C08B6.7	reproduction	.CGA[AC]G[AC]A[ACT]
C13G3.3	nucleotide binding	[AT]CG[AG]A[AGT]TA[ACT]
C14B1.7	nucleotide binding	[AT]CG[AG]A[AGT]TA[ACT]
C18E3.6	neuronal development	.[AG]ATCGAT[AT]
C27H6.4	gamete generation	[CGT]CG[AC]GA[AT][CG][AGT]
C47E8.8		[ACT][CT]GCG[GT][AC]C
CC8.2		.AACA[ACT]CG.
D1081.7	organ development	.[ACT]A[CT]GCGC.
D2030.6	organ development	.[ACT]A[CT]GCGC.
F12A10.8	neuronal development	.[AG]ATCGAT[AT]
F16A11.3	organ development	.[ACT]A[CT]GCGC.
F18A1.6	neuronal development	.[AG]ATCGAT[AT]
F19F10.11	organ development	.[ACT]A[CT]GCGC.
F25D7.4	nucleotide binding	[AT]CG[AG]A[AGT]TA[ACT]
F30F8.1	organ development	.[ACT]A[CT]GCGC.
F33G12.5	organ development	.[ACT]A[CT]GCGC.
F36D4.5	gamete generation	[CGT]CG[AC]GA[AT][CG][AGT]
F38A5.1	nucleotide binding	[AT]CG[AG]A[AGT]TA[ACT]
F39B2.11	nucleotide binding	[AT]CG[AG]A[AGT]TA[ACT]
F40F12.5	reproduction	.CGA[AC]G[AC]A[ACT]
F43D2.2		.AACA[ACT]CG
F44B9.4	organ development	.[ACT]A[CT]GCGC.
F44E2.8	neuronal development	.[AG]ATCGAT[AT]
F44E7.5	gamete generation	[CGT]CG[AC]GA[AT][CG][AGT]
F47D12.9	organ development	.[ACT]A[CT]GCGC.
F48E8.7	organ development	.[ACT]A[CT]GCGC.
F53C11.4	organ development	.[ACT]A[CT]GCGC.
F53F4.12	organ development	.[ACT]A[CT]GCGC.
F53H1.3	gamete generation	[CGT]CG[AC]GA[AT][CG][AGT]
F54A3.6	gamete generation	[CGT]CG[AC]GA[AT][CG][AGT]
F56A6.1	nucleotide binding	[AT]CG[AG]A[AGT]TA[ACT]
F56C9.10	gamete generation	[CGT]CG[AC]GA[AT][CG][AGT]

In order to assign GO-annotations to previously unannotated genes, the motif that was likely most functional in each gene was identified. Cluster and motif GO-annotations were considered when assigning new annotations, as was known function of the motif, if applicable. It is possible to separate the two distinct functions found in the larger cluster 7 through motif analysis, allowing suggested GO-annotations to be more specific. Some genes in the cluster either have no motifs identified through this analysis that are likely to be functional, or their most functional motif is not associated with any GO-term.

An additional 338 genes have a significant motif in their promoter region, but the genes with these motifs have no known coherent function.

## Discussion

In this study, we have identified the relative contribution of strain, stage, and strain by stage interaction on gene expression across development between two genetically divergent isolates of *C. elegans*. Over 90% of the genes were found to vary significantly over developmental time, and 71% of the genes were placed into clusters that represent patterns of differential expression over time. 20% of the genes display significant variation due to strain. A small but significant fraction of the genes display strain by stage interaction effects; that is, their pattern of expression over time differs between the two isolates.

### Much of the observed strain by stage variation is due to innate immune factors

Of the 283 genes with strain by stage variation, 58 have been directly implicated in innate immunity. Additional genes in this set belong to families involved in innate immunity, but the genes themselves have not been picked up in a screen. These genes are likely involved in innate immunity, but have not yet been identified because only a small subset of possible pathogens have ever been tested on *C. elegans* and the gene classes that have been implicated are among the fastest evolving gene families in the *C. elegans* genome [27], [30]. Because the *C. elegans* immune system is genetically hard-wired, large amounts of natural variation are needed in order to respond to the broad range of pathogens that a worm might encounter during its lifetime [66]. Since many of the gene families that are involved in innate immunity are evolving quickly, it may be that while the CB4856 allele is activated by pathogen exposure, the N2 allele is not, and thus the gene has not been identified as a member of the innate immune system.

Diverse expression patterns are observed in the genes that are involved in innate immunity; however, none of the genes are expressed at a high level during the egg stage. This could be because the eggshell provides better protection against pathogens than the cuticle of the larval and young adult worms, thus the embryonic worm does not have to commit resources to pathogen defense. Additionally, the major means of pathogenesis appears to be through ingestion [67], [68] and it is not until the larval stage that the worms begin to feed. Since they are not feeding, eggs may not have to protect against infection. Some of the genes are solely expressed in either N2 or in CB4856. Because the worms were not challenged by pathogens, it could be that these genes are expressed constitutively in one strain but are only expressed in response to the pathogen in the other. This is plausible, as some genes identified as involved in pathogen response in the N2 strain were not expressed in N2 during our time course. It is likely that we have identified new genes that are involved in innate immunity.

### Clustering and motif analysis allows for functional grouping of previously unknown genes and identification of novel motifs with a role in *C. elegans* development

Motif analysis of the 5′ promoter region and the 3′ UTR of the genes in each of the clusters led to the discovery of novel motifs that may have functional roles in development. Two novel motifs, [ACG][AG]CTTA[GT]A from the 5′ region and [AG]A[CT]A[AG]AT[CT] from the 3′ UTR, are enriched for the same GO-terms, namely structural constituent of the cuticle, ion/anion transport, and phosphate transport. Because 3′ UTRs tend to be targets for miRNAs, and miRNAs tend to be negative regulators of their targets, it is possible that these two motifs represent a mechanism for both positive and negative regulation of the same process.

Uncovering the phenotype of previously identified miRNAs is an open field, as the identification of miRNAs through sequencing has outpaced the study of their function. In our analysis we identified the binding site for miR-238/239. Currently, there is no known function for these miRNAs. However, their targets are enriched for receptor activity and neurotransmitter binding. It has been previously shown that miRNAs are responsible for at least one case of left/right patterning in the *C. elegans* nervous system [69]. It is likely that these miRNAs are also responsible for regulating neural differentiation.

Much of the *C. elegans* genome is unannotated with regard to function or GO-term category. In our set of roughly 9,000 genes that cluster using the STEM algorithm, over 2,500 have no known function or GO-annotation. Although annotation using small scale, directed experiments is often more accurate that using large scale data, many of the unannotated genes will likely have no obvious function or their function would have been identified in one of the many RNAi screens in *C. elegans*. By combining clustering with motif analysis we were able to separate the function of large clusters, which should provide a more accurate annotation for these genes. We have proposed general GO-terms for 1,568 previously unannotated genes.

This work provides new insights into the type of genes that differ between natural isolates of *C. elegans*. Many of the genes identified belong to the innate immune system. Because the innate immune system is hard-wired, genetic diversity must be present within the species to allow for the varying pathogen exposures based on environment. As these genes are expressed even in the absence of the pathogen, they may also serve another developmental function. In addition, we show that by combining clustering with motif discovery, biological coherence of clusters can be increased, aiding large-scale annotation efforts.

## Materials and Methods

### Strains and Maintenance

Wild-type N2 (Bristol) and CB4856 (Hawaii) worms were obtained from the *Caenorhabditis* Genetics Center (University of Minnesota, Minneapolis, MN). Strains were maintained according to established procedures [70] and all experiments were carried out at 20°C.

### Synchronization

Hermaphrodite-only worms were grown on 10 cm plates of Nematode Growth Medium agar (NGM) seeded with 1 mL of OP50 and kept at a constant temperature of 20°C. Care was taken to ensure that worms on the plates remained unstarved for more than three generations before using the populations for RNA extraction. Worms were synchronized as previously described [8]. Developmental stage was ascertained through the appearance of the gonad, as well as the size of the worm and the time post-hatching [71]. Each larval stage was assayed roughly halfway through the stage. Young adults were collected at the time that the first egg was laid on the plate and the eggs were collected at this time as well, so that while not synchronized they should all be young embryos [8]. In order to minimize the effect of starvation, L1 worms were collected 5 hours after being plated on OP50. Six time points per strain were present in the final dataset: egg, L1, L2, L3, L4 and young adult.

### RNA Isolation

The protocol was adapted from [10]. Briefly, at the correct developmental time point, the synchronized worms were washed in M9 and sucrose floated. Trizol (Invitrogen, Carlsbad, CA) was added and the worms were subjected to a freeze/thaw cycle. RNA was isolated using chloroform and phase-lock tubes (Invitrogen, Carlsbad, CA), precipitated using isopropanol and cleaned using an RNeasy kit (Qiagen, Valencia CA). RNA quality was checked using the Nano-drop ND-1000 UV-Vis spectrophotometer and some samples were checked using a bioanalyzer (Agilent, San Jose CA).

### Labeling and Hybridization

RNA was labeled using the Low RNA Input Fluorescent Linear Amplification Kit (Agilent, San Jose CA) according to the manufacturer's instructions. The reference used is a 50∶50 N2∶CB4856 combination of RNA isolated from separate plates of hermaphrodite only mixed-stage populations. Four replicates of each time point were completed. For each time point two of the experimental samples were labeled with Cy3 while two were labeled with Cy5. 850 ng of an experimental sample and of the reference were hybridized to Agilent *C. elegans* 4×44k oligo microarrays for each array, according to the manufacturer's protocol. Samples were loaded into each array randomly on the slide, so that each slide did not contain more than one sample from each time point. The slides were scanned using an Agilent DNA microarray scanner and the data was extracted using Agilent Feature Extractor (version 9.5).

### Microarray normalization, filtering, and analysis

The array data was uploaded to the Princeton University Microarray Database (PUMAdb) for processing. The data was collapsed by SUID, using the average value of each probe. Spots were considered good data if intensity was well above background and the feature was not a nonuniformity outlier Only genes with greater than 80% good data were kept for further analysis. Missing values were imputed using KNN-impute in the MultiExperiment Viewer [72], [73]. All array data will be made publicly available through puma.princeton.edu. For the analysis using only a single stage, 100% good data was required across all arrays for that time point. Data was hierarchically clustered by centroid linkage and a centered Pearson correlation using the average value from the four replicates [17]. Clusters were visualized using JavaTreeView [74]. An analysis of variance (ANOVA) test was used to see which genes changed significantly over developmental time, using the strain and stage of each array as the parameters. qvalue, an R-package, was used to obtain false-discovery rates (FDR) [75]. Significant genes have an FDR of less than .05. Enrichment values for functional groups were calculated from lists of genes provided by WormBase [76], which uses the PFAM and Interpro databases. The list of innate immunity genes was hand-curated from the literature. Significance was assessed through the hypergeometric distribution. Short-Time series expression miner (STEM) was used to cluster the data and provide GO-term enrichments [33]. FIRE was used for motif analysis [38]. The best motif for each gene was defined as that motif with the highest pa+po+pd value. This value had to be greater than 4 in order for a motif to be considered real. The most recent *C. elegans* gene ontology was downloaded from the Gene Ontology Consortium on April 20^th^, 2008 [77]. The assignment of function was done subjectively, using GO-enrichments of the clusters, the motifs, and previous knowledge about the named motifs.

## Supporting Information

Table S1 Supplementary table 1
(0.06 MB XLS)

Table S2 Supplementary table 2
(0.06 MB DOC)

Table S3 Supplementary table 3
(0.12 MB DOC)

Table S4 Supplementary table 4
(0.37 MB DOC)

Table S5 Supplementary table 5
(0.18 MB XLS)

Table S6 Supplementary table 6
(0.24 MB XLS)
