# Supplementary material for: Comparative Developmental Expression Profiling of Two C. elegans Isolates
Source: PLoS One. 2008 Dec 31;3(12):e4055. doi: 10.1371/journal.pone.0004055 (PMC2605249; doi:10.1371/journal.pone.0004055)
Supplement: Table S2 — Supplementary table 2 (0.06 MB DOC) [file pone.0004055.s002.doc]

| **Cluster B (CB4856)** | **Cluster B (N2 )** | **Cluster C** | | **Cluster D** |
| --- | --- | --- | --- | --- |
| ZK1290.10  T10E9.3  F45F10.13  C36C5.15  C36C5.14  Y53F4B.32  R09A8.4  ZK617.2  C14C6.12  C35D6.5  K06H6.2  K06H6.1  C14C6.3  R11G11.14  Y54G2A.8  T13F3.9  F38A1.6  F55G11.6  F55G11.7  C39B5.10  Y39B6A.21  ZK1240.9  M02D8.5  C09E9.2  Y17D7A.1  C32H11.9  C04G6.1 | Y69H2.9  C29F9.6  Y82E9BR.21  F15E11.15  T19H12.8  F36H5.2  T07D3.4  Y111B2A.21  T17A3.8  K07E8.3  C10H11.3  C29F9.4  C40D2.2  C29F9.2  C04E12.2  F35F10.4  Y82E9BL.14  Y40D12A.2  F53C3.13  Y57A10B.3  Y53F4B.30  F54E2.1  F39E9.2  M04C3.3  T04A8.13  T12B5.11  F57E7.1  Y102A5B.3  F35E8.11  T10H4.11 | F32B5.3  F28A10.5  F57A10.2  C07E3.4  Y54G2A.13  F48F5.1  Y38F1A.1  K08C9.2  Y39G10AR.16  C06A5.2  F08G2.6  C35E7.10  C55C3.4  C47D12.3  F59B2.5  K07A1.14  R09E10.1  Y38E10A.17  F23C8.7  C46E10.1  F35C11.2  K07F5.4  C25D7.12  Y62H9A.12  C17H12.5  C17H12.3  M88.3  C04G2.2  F19B6.4  C39H7.1  Y38H8A.3  C38C3.3  F47D12.7  F53B6.4  W02B12.12  Y43F8A.2  T01C3.5  W09C3.6  T27A3.5  K06A5.2  F44D12.4  F07A5.2  C35D10.2  ZK945.7  C27D8.1  F36H12.8  C25G4.6  BE10.1  C30B5.3  M04G7.2  ZK616.7  Y69E1A.2  C09B9.2  ZC317.6  C09D4.3  H06H21.9 | ZC513.3  Y69A2AR.19  ZK1290.6  M70.3  R155.2  F36H12.3  F35E2.9  F58D2.2  C35E7.9  K01A11.4  Y57G7A.6  T08B6.4  ZK1010.5  T10E9.4  W03F11.4  ZK809.1  C50F4.2  ZK757.3  T22B3.2  C24D10.1  T15B12.2  ZK507.3  K03H1.1  H04M03.1  W03D8.2  ZK973.8  C29F5.3  C53A5.4  K10C9.7  ZC412.7  C40H5.1  F46A8.6  T25B9.6  F25H5.7  F52H3.6  ZK938.1  H06O01.4  C27D8.2  K04H4.5  R07E5.6  C10G11.8  F57F4.1  Y8G1A.1  R08A2.1  C25A8.5  Y113G7C.1  W03D8.9  R13H9.5  F13A7.1  F54C1.9  F53C3.1  Y106G6G.1  ZK1053.6  ZK520.5  ZC116.2 | Y38E10A.10  Y46C8AL.5  Y46C8AL.4  F45E4.1  F35C5.12  F35F10.6  T28A11.16  C29F3.7  W07B8.1  Y37D8A.6  C54D10.1  D1054.8  W04E12.7  W04E12.7  F16H6.7  F35E8.8  T06C12.10  Y82E9BL.11  Y82E9BL.10  F59A6.6  F08G5.6  D2085.7  F15A4.6  W06B3.1  C46F9.1  Y39G10AR.6  C47B2.6 |

Table S2. Gene lists for clusters shown in Figure 2

Genes displaying significant strain by stage variation were clustered as described in the figure legend.
