# Supplementary material for: Comparative Developmental Expression Profiling of Two C. elegans Isolates
Source: PLoS One. 2008 Dec 31;3(12):e4055. doi: 10.1371/journal.pone.0004055 (PMC2605249; doi:10.1371/journal.pone.0004055)
Supplement: Table S3 — Supplementary table 3 (0.12 MB DOC) [file pone.0004055.s003.doc]

Table S3. Significant GO-terms in the STEM clusters

Significant GO-term enrichment (p<.01 after correction) for significant STEM clusters using all time points, including the young adult. GO-term are listed in decreasing order of significance.

| **Cluster 1** |
| --- |
| intrinsic to membrane  integral to membrane  membrane part  membrane  2,3-dihydro-2,3-dihydroxybenzoate dehydrogenase activity  enterobactin biosynthetic process  enterobactin metabolic process  catechol metabolic process  siderophore biosynthetic process from catechol  oxidoreductase activity, acting on the CH-CH group of donors, NAD or NADP as acceptor  siderophore metabolic process  siderophore biosynthetic process  phenol metabolic process  ribosome  structural constituent of ribosome  cell part  rhodopsin-like receptor activity  transmembrane receptor activity  receptor activity |
| **Cluster 2** |
| multicellular organismal development  embryonic development ending in birth or egg hatching  embryonic development  nucleic acid binding  positive regulation of growth rate  regulation of growth rate  anatomical structure development  positive regulation of growth  regulation of growth  positive regulation of biological process  post-embryonic development  larval development (sensu Nematoda)  helicase activity  larval development  genitalia development  organ development  hermaphrodite genitalia development  reproductive process  regulation of biological process  sex differentiation  system development  reproductive developmental process  RNA processing  ATP-dependent helicase activity  anatomical structure morphogenesis  nucleotide binding  DNA metabolic process  nucleobase, nucleoside, nucleotide and nucleic acid metabolic process  intracellular  nucleoside-triphosphatase activity  gene silencing  biopolymer metabolic process  ATPase activity  pyrophosphatase activity  hydrolase activity, acting on acid anhydrides, in phosphorus-containing anhydrides  hydrolase activity, acting on acid anhydrides  ATPase activity, coupled  ATP binding  adenyl ribonucleotide binding  nuclease activity  ribonucleotide binding  purine ribonucleotide binding  nucleus  adenyl nucleotide binding  cell cycle  purine nucleotide binding  RNA-mediated posttranscriptional gene silencing  posttranscriptional gene silencing  RNA-mediated gene silencing  cell cycle process  RNA interference  regulation of gene expression, epigenetic  morphogenesis of an epithelium  hydrolase activity  protein binding  meiotic cell cycle  body morphogenesis  cell division  nucleotidyltransferase activity  nuclear part  organelle organization and biogenesis  meiosis  M phase of meiotic cell cycle  M phase  chromosome  double-stranded RNA binding  membrane-bound organelle  small protein conjugating enzyme activity  ubiquitin-protein ligase activity  cell cycle phase  intracellular membrane-bound organelle  macromolecule metabolic process  tRNA processing  ubiquitin cycle  reproductive process in a multicellular organism  multicellular organism reproduction  gastrulation with mouth forming first  oviposition  reproductive behavior in a multicellular organism  cytokinesis  organelle part  RNA binding  intracellular organelle  reproductive behavior  intracellular organelle part  chromatin modification  chromosome segregation  gastrulation  DNA recombination |
| **Cluster 3** |
| embryonic development ending in birth or egg hatching  embryonic development  P granule  pole plasm  transcription initiation  multicellular organismal development  RNA polymerase II transcription factor activity  nuclear part  DNA-directed RNA polymerase II, holoenzyme  protein-DNA complex assembly  transcription initiation from RNA polymerase II promoter  cell cycle phase  M phase |
| **Cluster 4** |
| anatomical structure development  reproductive process  multicellular organismal development  embryonic development  regulation of biological process  embryonic development ending in birth or egg hatching  system development  organ development  anatomical structure morphogenesis  reproductive developmental process  intracellular  nucleus  sex differentiation  larval development (sensu Nematoda)  larval development  post-embryonic development  membrane-bound organelle  genitalia development  intracellular membrane-bound organelle  hermaphrodite genitalia development  nucleic acid binding  intracellular organelle  biopolymer metabolic process  regulation of cellular process  positive regulation of biological process  intracellular part  cellular component organization and biogenesis  regulation of growth  mitotic cell cycle  positive regulation of growth  morphogenesis of an epithelium  regulation of growth rate  macromolecule metabolic process  organelle organization and biogenesis  DNA binding  positive regulation of growth rate  protein binding  vulval development  body morphogenesis  regulation of gene expression  cell cycle  microtubule-based process  microtubule cytoskeleton organization and biogenesis  chromosome  intracellular signaling cascade  cell differentiation  spindle organization and biogenesis  mitotic spindle organization and biogenesis  organelle part  cell cycle process  cellular developmental process  intracellular organelle part  regulation of nucleobase, nucleoside, nucleotide and nucleic acid metabolic process  transcription  nucleobase, nucleoside, nucleotide and nucleic acid metabolic process  small GTPase mediated signal transduction  regulation of metabolic process  regulation of cellular metabolic process  cytoskeleton organization and biogenesis  regulation of transcription  primary metabolic process  regulation of small GTPase mediated signal transduction  negative regulation of biological process  biopolymer modification  regulation of vulval development  RNA biosynthetic process  GTPase regulator activity  nuclear membrane part  nuclear part  cellular metabolic process  transcription, DNA-dependent  signal transduction  regulation of developmental process  establishment of cellular localization  RNA metabolic process  nuclear membrane  nucleotide binding  protein modification process  regulation of signal transduction  cellular localization  establishment of organelle localization  protein complex  organelle localization  regulation of transcription, DNA-dependent  Ras protein signal transduction  nucleus localization  nuclear migration  establishment of nucleus localization  cell communication  dosage compensation  single fertilization  fertilization  chromosomal part  negative regulation of vulval development  reproductive process in a multicellular organism  oviposition  reproductive behavior in a multicellular organism  multicellular organism reproduction  GTPase activator activity  regulation of Ras protein signal transduction  dosage compensation, by hypoactivation of X chromosome  chromosome organization and biogenesis  reproductive behavior  pronuclear migration  protein-tyrosine kinase activity  protein localization  negative regulation of developmental process  small GTPase regulator activity  cell fate commitment  cell division  post-translational protein modification  macromolecule localization  nuclear envelope  behavior  regulation of transcription from RNA polymerase II promoter  transcription from RNA polymerase II promoter  cell cortex  regulation of locomotion  organ morphogenesis  DNA replication initiation  intracellular non-membrane-bound organelle  non-membrane-bound organelle  DNA metabolic process |
| **Cluster 5** |
| nucleosome assembly  nucleosome  chromatin assembly  protein-DNA complex assembly  chromatin assembly or disassembly  DNA packaging  establishment and/or maintenance of chromatin architecture  chromatin  macromolecular complex assembly  chromosome organization and biogenesis  mRNA metabolic process  embryonic development  cellular component assembly  embryonic development ending in birth or egg hatching  chromosomal part  multicellular organismal development  chromosome  cellular component organization and biogenesis  threonine endopeptidase activity  proteasome core complex (sensu Eukaryota) |
| **Cluster 6** |
| ribosome  structural constituent of ribosome  ribonucleoprotein complex  translation  macromolecule biosynthetic process  cytoplasmic part  cellular biosynthetic process  intracellular non-membrane-bound organelle  non-membrane-bound organelle  biosynthetic process  cytoplasm  sugar binding  carbohydrate binding  pepsin A activity  cellular protein metabolic process  cellular macromolecule metabolic process  aspartic-type endopeptidase activity  protein metabolic process  ribosomal subunit  intracellular organelle  intracellular part  peptidase activity  large ribosomal subunit  primary metabolic process  proteolysis  fatty acid biosynthetic process  organic acid biosynthetic process  carboxylic acid biosynthetic process  cellular metabolic process  endopeptidase activity |
| **Cluster 7** |
| embryonic development ending in birth or egg hatching  embryonic development  multicellular organismal development  cell cycle process  cell cycle  cell division  cell cycle phase  nucleotide binding  M phase  ribonucleotide binding  purine ribonucleotide binding  meiosis  M phase of meiotic cell cycle  adenyl ribonucleotide binding  cytokinesis  purine nucleotide binding  meiotic cell cycle  ATP binding  adenyl nucleotide binding  response to DNA damage stimulus  response to endogenous stimulus  cellular component organization and biogenesis  sexual reproduction  intracellular  DNA repair  chromosome segregation  cytoskeleton organization and biogenesis  regulation of cell cycle  DNA metabolic process  organelle organization and biogenesis  protein catabolic process  microtubule-based process  intracellular part  macromolecule catabolic process  reproductive developmental process  reproductive process  meiotic chromosome segregation  biopolymer catabolic process  mitotic cell cycle  biopolymer metabolic process  spindle organization and biogenesis  gamete generation  establishment of organelle localization  organelle localization  establishment of cellular localization  microtubule cytoskeleton organization and biogenesis  sex differentiation  hermaphrodite genitalia development  organelle part  genitalia development  cellular localization  nucleoside-triphosphatase activity  microtubule cytoskeleton  intracellular organelle part  response to stress  system development  intracellular organelle  primary metabolic process  anatomical structure development  pyrophosphatase activity  hydrolase activity, acting on acid anhydrides, in phosphorus-containing anhydrides  catabolic process  cell cortex  hydrolase activity, acting on acid anhydrides  organ development  chromosome  modification-dependent protein catabolic process  ubiquitin-dependent protein catabolic process  modification-dependent macromolecule catabolic process  proteolysis involved in cellular protein catabolic process  macromolecule metabolic process  cellular protein catabolic process  DNA-dependent DNA replication  cytoskeletal part  ubiquitin-specific protease activity  small conjugating protein-specific protease activity  mitotic spindle organization and biogenesis  cytoplasm  cellular metabolic process  cellular macromolecule catabolic process  helicase activity  larval development (sensu Nematoda)  larval development  mismatched DNA binding  maintenance of fidelity during DNA-dependent DNA replication  mismatch repair  centrosome  post-embryonic development  intracellular non-membrane-bound organelle  non-membrane-bound organelle  microtubule organizing center  protein complex |
| **Cluster 8** |
| cell communication  signal transducer activity  signal transduction  substrate specific channel activity  ion channel activity  channel activity  passive transmembrane transporter activity  receptor activity  transmembrane receptor activity  cell surface receptor linked signal transduction  neuron projection  cell projection  G-protein coupled receptor protein signaling pathway  gated channel activity  ion transmembrane transporter activity  G-protein coupled receptor activity  transmembrane transporter activity  substrate-specific transmembrane transporter activity  cation channel activity  substrate-specific transporter activity  rhodopsin-like receptor activity  metal ion transmembrane transporter activity  ion transport  axon  extracellular ligand-gated ion channel activity  neurotransmitter receptor activity  neurotransmitter binding  synapse part  intracellular signaling cascade  ligand-gated ion channel activity  ligand-gated channel activity  metal ion transport  cell soma  cell part  membrane part  extracellular-glutamate-gated ion channel activity  excitatory extracellular ligand-gated ion channel activity  cation transport  feeding behavior  cation transmembrane transporter activity  integral to membrane  hyperosmotic response  response to osmotic stress  intrinsic to membrane  response to abiotic stimulus  postsynaptic membrane  membrane  glutamate receptor activity  neurological system process  cell-cell signaling  potassium ion transport  transmission of nerve impulse  phosphorus-oxygen lyase activity  cyclic nucleotide metabolic process  cyclic nucleotide biosynthetic process  potassium channel activity  regulation of pharyngeal pumping  monovalent inorganic cation transport  calcium channel activity  establishment of localization  transport  guanylate cyclase activity  cGMP metabolic process  ionotropic glutamate receptor activity  cGMP biosynthetic process  voltage-gated channel activity  voltage-gated ion channel activity  cyclase activity  behavior  calcium ion transport  pharyngeal pumping  eating behavior  voltage-gated cation channel activity  synaptic transmission  response to external stimulus  system process  dendrite  di-, tri-valent inorganic cation transport |
| **Cluster 9** |
| monooxygenase activity  transmembrane transporter activity  oxidoreductase activity  substrate specific channel activity  ion channel activity  channel activity  passive transmembrane transporter activity  substrate-specific transporter activity  substrate-specific transmembrane transporter activity  basement membrane  ion transmembrane transporter activity  metal ion transport  monovalent inorganic cation transport  cation channel activity  ion transport  serine hydrolase activity  serine-type peptidase activity  integral to membrane  intrinsic to membrane  iron ion binding  extracellular matrix part  tetrapyrrole binding  heme binding  membrane part |
| **Cluster 10** |
| regulation of transcription, DNA-dependent  transcription factor activity  transcription, DNA-dependent  regulation of cellular process  RNA biosynthetic process  sequence-specific DNA binding  regulation of transcription  regulation of nucleobase, nucleoside, nucleotide and nucleic acid metabolic process  regulation of gene expression  regulation of cellular metabolic process  transcription  zinc ion binding  regulation of metabolic process  nucleic acid binding  RNA metabolic process  DNA binding  cation binding  transition metal ion binding  metal ion binding  biopolymer metabolic process  ion binding  nucleus  regulation of biological process  system development  intracellular  protein binding  organ development  cell migration  membrane-bound organelle  localization of cell  cell motility  intracellular membrane-bound organelle |
| **Cluster 11** |
| integral to membrane  intrinsic to membrane  membrane part  membrane  active transmembrane transporter activity  ion transport  transport  ATPase activity, coupled to transmembrane movement of substances  ATPase activity, coupled to movement of substances  establishment of localization  hydrolase activity, acting on acid anhydrides, catalyzing transmembrane movement of substances  P-P-bond-hydrolysis-driven transmembrane transporter activity  primary active transmembrane transporter activity  transmembrane transporter activity  anion transport  structural constituent of cuticle  inorganic anion transport  phosphate transport  astacin activity  metallopeptidase activity  ion transmembrane transporter activity  cell part |
| **Cluster 12** |
| phosphate transport  inorganic anion transport  anion transport  structural constituent of cuticle  ion transport  intein-mediated protein splicing  protein splicing  protein processing  cytoplasm  transport  establishment of localization |
| **Cluster 13** |
| signal transducer activity  receptor activity  integral to membrane  intrinsic to membrane  membrane part  membrane  cell part  steroid hormone receptor activity  ligand-dependent nuclear receptor activity  neurotransmitter receptor activity  neurotransmitter binding  extracellular ligand-gated ion channel activity  synapse part  sequence-specific DNA binding  ligand-gated channel activity  ligand-gated ion channel activity  transcription factor activity  substrate specific channel activity  ion channel activity  channel activity  passive transmembrane transporter activity  postsynaptic membrane  transmembrane receptor activity  gated channel activity  regulation of transcription, DNA-dependent  cell communication  G-protein coupled receptor protein signaling pathway  transcription, DNA-dependent  RNA biosynthetic process  regulation of transcription  G-protein coupled receptor activity  cell surface receptor linked signal transduction  rhodopsin-like receptor activity  regulation of nucleobase, nucleoside, nucleotide and nucleic acid metabolic process  regulation of cellular metabolic process  signal transduction  transcription  regulation of gene expression  regulation of metabolic process  ion transmembrane transporter activity |
