# Supplementary material for: Comparative Developmental Expression Profiling of Two C. elegans Isolates
Source: PLoS One. 2008 Dec 31;3(12):e4055. doi: 10.1371/journal.pone.0004055 (PMC2605249; doi:10.1371/journal.pone.0004055)
Supplement: Table S4 — Supplementary table 4 (0.37 MB DOC) [file pone.0004055.s004.doc]

Table S4. Significant GO-terms for targets of motifs identified by FIRE

FIRE searched the targets of the motifs defined for GO-enrichment values. STEM clusters were used as FIRE input; FIRE was run both with and without the young adult time point. Only STEM clusters that we significantly enriched for GO-terms associated with development were used for the egg-L4 dataset. Listed are all significant motifs that had one or more significant GO-terms associated with their targets. 5’ and 3’UTR motifs are listed separately.

| **Motifs from the 5’ end identified in the complete dataset** | | |
| --- | --- | --- |
| .[ACT]A[CT]GCGC. | 2.33E-06 | reproduction |
| .[ACT]A[CT]GCGC. | 2.51E-06 | genitalia development |
| .[ACT]A[CT]GCGC. | 2.51E-06 | hermaphrodite genitalia development |
| .[ACT]A[CT]GCGC. | 3.22E-06 | organ development |
| .[ACT]A[CT]GCGC. | 5.87E-06 | sex differentiation |
| .[ACT]A[CT]GCGC. | 1.34E-05 | response to stimulus |
| .[ACT]A[CT]GCGC. | 8.29E-05 | nucleotide binding |
| .[ACT]A[CT]GCGC. | 4.65E-04 | behavior |
| .[ACT]A[CT]GCGC. | 6.35E-04 | locomotory behavior |
| .[ACT]A[CT]GCGC. | 9.61E-04 | protein modification |
| .[ACT]A[CT]GCGC. | 2.28E-03 | biopolymer modification |
| .[ACT]A[CT]GCGC. | 3.92E-03 | ubiquitin cycle |
| .[AG]ATCGAT[AT] | 1.90E-05 | reproduction |
| .[AG]ATCGAT[AT] | 7.07E-05 | locomotory behavior |
| .[AG]ATCGAT[AT] | 2.38E-04 | biopolymer modification |
| .[AG]ATCGAT[AT] | 9.11E-04 | protein modification |
| .[AG]ATCGAT[AT] | 1.21E-03 | ubiquitin cycle |
| .[AG]ATCGAT[AT] | 2.06E-03 | nucleotide binding |
| .[AG]ATCGAT[AT] | 2.65E-03 | response to stimulus |
| .[AG]ATCGAT[AT] | 4.55E-03 | behavior |
| [AG]A[AGT]CGC[AT][CG]. | 7.51E-03 | intracellular organelle part |
| [AG]A[AGT]CGC[AT][CG]. | 7.51E-03 | organelle part |
| [CGT]CG[AC]GA[AT][CG][AGT] | 2.59E-05 | reproduction |
| [CGT]CG[AC]GA[AT][CG][AGT] | 5.24E-05 | gametogenesis |
| [CGT]CG[AC]GA[AT][CG][AGT] | 1.15E-04 | sexual reproduction |
| [CGT]CG[AC]GA[AT][CG][AGT] | 3.14E-03 | helicase activity |
| .C[CT]TATCA. | 1.36E-09 | sugar binding |
| .C[CT]TATCA. | 2.16E-08 | carbohydrate binding |
| [CT]AG[GT][CT]AG[AGT][CGT] | 1.25E-03 | structural constituent of cuticle |
| [CT]AG[GT][CT]AG[AGT][CGT] | 1.28E-03 | phosphate transport |
| [CT]AG[GT][CT]AG[AGT][CGT] | 2.94E-03 | inorganic anion transport |
| [CT]AG[GT][CT]AG[AGT][CGT] | 4.03E-03 | anion transport |
| .TTTCAAA. | 4.20E-16 | transporter activity |
| .TTTCAAA. | 1.17E-15 | integral to membrane |
| .TTTCAAA. | 1.17E-15 | intrinsic to membrane |
| .TTTCAAA. | 8.28E-15 | membrane part |
| .TTTCAAA. | 1.31E-11 | receptor activity |
| .TTTCAAA. | 3.07E-10 | signal transducer activity |
| .TTTCAAA. | 1.84E-09 | transmembrane receptor activity |
| .TTTCAAA. | 3.75E-09 | ion transporter activity |
| .TTTCAAA. | 2.89E-08 | ion channel activity |
| .TTTCAAA. | 2.89E-08 | alpha-type channel activity |
| .TTTCAAA. | 2.89E-08 | channel or pore class transporter activity |
| .TTTCAAA. | 1.82E-06 | cation transporter activity |
| .TTTCAAA. | 3.14E-06 | metal ion transport |
| .TTTCAAA. | 4.88E-06 | ion transport |
| .TTTCAAA. | 5.01E-06 | cation channel activity |
| .TTTCAAA. | 7.18E-06 | G-protein coupled receptor activity |
| .TTTCAAA. | 1.15E-05 | potassium ion transport |
| .TTTCAAA. | 1.17E-05 | oxidoreductase activity |
| .TTTCAAA. | 1.55E-05 | monovalent inorganic cation transport |
| .TTTCAAA. | 1.97E-05 | cation transport |
| .TTTCAAA. | 3.09E-05 | potassium channel activity |
| .TTTCAAA. | 6.55E-05 | G-protein coupled receptor protein |
| .TTTCAAA. | 8.17E-05 | tetrapyrrole binding |
| .TTTCAAA. | 8.17E-05 | heme binding |
| .TTTCAAA. | 1.59E-04 | rhodopsin-like receptor activity |
| .TTTCAAA. | 3.15E-04 | carrier activity |
| .TTTCAAA. | 4.64E-04 | cell surface receptor linked signal transduction |
| .TTTCAAA. | 5.24E-03 | monooxygenase activity |
| .A[CG][ACT]TAAG[ACT] | 4.46E-03 | rhodopsin-like receptor activity |
| [AT]AAG[AGT]TCA. | 1.84E-05 | integral to membrane |
| [AT]AAG[AGT]TCA. | 1.84E-05 | intrinsic to membrane |
| [AT]AAG[AGT]TCA. | 1.60E-04 | membrane part |
| [AT]AAG[AGT]TCA. | 4.06E-04 | oxidoreductase activity |
| [AT]AAG[AGT]TCA. | 2.31E-03 | G-protein coupled receptor |
| [ACT]A[CG]TA[CGT]AC[ACT] | 1.66E-03 | signal transducer activity |
| [ACT]A[CG]TA[CGT]AC[ACT] | 7.38E-03 | receptor activity |
| [ACT]TT[GT]ATAC. | 4.14E-15 | phosphate transport |
| [ACT]TT[GT]ATAC. | 2.31E-14 | inorganic anion transport |
| [ACT]TT[GT]ATAC. | 4.47E-14 | anion transport |
| [ACT]TT[GT]ATAC. | 7.98E-14 | structural constituent of cuticle |
| [ACT]TT[GT]ATAC. | 6.30E-07 | ion transport |
| [ACT]TT[GT]ATAC. | 1.15E-06 | structural molecule activity |
| [ACT]TT[GT]ATAC. | 7.21E-06 | cytoplasm |
| [CGT][ACG]ACC[CT]A[CG][ACT] | 1.70E-03 | signal transducer activity |
| [CGT][ACG]ACC[CT]A[CG][ACT] | 8.71E-03 | receptor activity |
| [ACG][CT]CT[AT]A[GT]A. | 6.61E-06 | transmembrane receptor activity |
| [ACG][CT]CT[AT]A[GT]A. | 1.37E-05 | rhodopsin-like receptor activity |
| [ACG][CT]CT[AT]A[GT]A. | 1.48E-05 | signal transducer activity |
| [ACG][CT]CT[AT]A[GT]A. | 2.58E-05 | G-protein coupled receptor protein signaling pathway |
| [ACG][CT]CT[AT]A[GT]A. | 2.62E-05 | receptor activity |
| [ACG][CT]CT[AT]A[GT]A. | 3.59E-05 | G-protein coupled receptor activity |
| [ACG][CT]CT[AT]A[GT]A. | 2.27E-04 | cell surface receptor linked signal transduction |
| [ACG][CT]CT[AT]A[GT]A. | 4.25E-04 | integral to membrane |
| [ACG][CT]CT[AT]A[GT]A. | 4.25E-04 | intrinsic to membrane |
| [ACG][CT]CT[AT]A[GT]A. | 2.68E-03 | membrane part |
| [AT]C.TAT[ACG]C[AT] | 5.35E-09 | anion transport |
| [AT]C.TAT[ACG]C[AT] | 1.85E-08 | inorganic anion transport |
| [AT]C.TAT[ACG]C[AT] | 1.26E-07 | ion transport |
| [AT]C.TAT[ACG]C[AT] | 1.69E-07 | phosphate transport |
| [AT]C.TAT[ACG]C[AT] | 4.30E-06 | structural constituent of cuticle |
| .TTTTGAC[ACT] | 4.03E-09 | membrane part |
| .TTTTGAC[ACT] | 4.19E-09 | integral to membrane |
| .TTTTGAC[ACT] | 4.19E-09 | intrinsic to membrane |
| .TTTTGAC[ACT] | 9.45E-05 | transporter activity |
| .TTTTGAC[ACT] | 1.61E-03 | rhodopsin-like receptor activity |
| .TTTTGAC[ACT] | 4.00E-03 | receptor activity |
| .TTTTGAC[ACT] | 4.12E-03 | G-protein coupled receptor protein signaling pathway |
| .TTTTGAC[ACT] | 5.27E-03 | cell surface receptor linked signal transduction |
| [ACT]ACCAAAA. | 2.65E-03 | membrane part |
| .AC[AG]TCAT. | 3.20E-04 | neuropeptide signaling pathway |
| .AC[AG]TCAT. | 1.54E-03 | G-protein coupled receptor protein signaling pathway |
| .AC[AG]TCAT. | 6.60E-03 | cell surface receptor linked signal transduction |
| .CGA[AC]G[AC]A[ACT] | 3.30E-03 | reproduction |
| .CGA[AC]G[AC]A[ACT] | 6.20E-03 | pyrophosphatase activity |
| .CGA[AC]G[AC]A[ACT] | 6.20E-03 | hydrolase activity, acting on acid anhydrides |
| .CGA[AC]G[AC]A[ACT] | 6.20E-03 | hydrolase activity, acting on acid anhydrides, in phosphorus-containing anhydrides |
| .CGA[AC]G[AC]A[ACT] | 9.18E-03 | double-stranded RNA binding |
| .A.CGGAG[ACT] | 1.61E-03 | helicase activity |
| [AT]CG[AG]A[AGT]TA[ACT] | 2.70E-07 | nucleotide binding |
| [AT]CG[AG]A[AGT]TA[ACT] | 5.97E-05 | purine nucleotide binding |
| [AT]CG[AG]A[AGT]TA[ACT] | 8.30E-05 | ATP binding |
| [AT]CG[AG]A[AGT]TA[ACT] | 1.23E-04 | adenyl nucleotide binding |
| [AT]CG[AG]A[AGT]TA[ACT] | 2.10E-04 | DNA metabolism |
| [AT]CG[AG]A[AGT]TA[ACT] | 3.56E-04 | pyrophosphatase activity |
| [AT]CG[AG]A[AGT]TA[ACT] | 3.56E-04 | hydrolase activity, acting on acid anhydrides |
| [AT]CG[AG]A[AGT]TA[ACT] | 3.56E-04 | hydrolase activity, acting on acid anhydrides |
| [AT]CG[AG]A[AGT]TA[ACT] | 6.82E-04 | nucleoside-triphosphatase activity |
| [CGT][ACG]A[CT]AGTA[AGT] | 2.97E-06 | ion transport |
| [CGT][ACG]A[CT]AGTA[AGT] | 1.09E-04 | anion transport |
| [CGT][ACG]A[CT]AGTA[AGT] | 6.93E-04 | inorganic anion transport |
| .TTGCCA[AC][ACT] | 1.58E-04 | receptor activity |
| .TTGCCA[AC][ACT] | 3.53E-04 | signal transducer activity |
| .TCAT[AT]AC. | 1.42E-15 | anion transport |
| .TCAT[AT]AC. | 1.26E-14 | inorganic anion transport |
| .TCAT[AT]AC. | 2.33E-14 | structural constituent of cuticle |
| .TCAT[AT]AC. | 7.43E-13 | phosphate transport |
| .TCAT[AT]AC. | 7.57E-10 | ion transport |
| .TCAT[AT]AC. | 8.19E-07 | structural molecule activity |
| .TCAT[AT]AC. | 1.94E-04 | cytoplasm |
| [ACG][AG]CTTA[GT]A. | 2.89E-11 | phosphate transport |
| [ACG][AG]CTTA[GT]A. | 5.67E-11 | inorganic anion transport |
| [ACG][AG]CTTA[GT]A. | 7.36E-11 | anion transport |
| [ACG][AG]CTTA[GT]A. | 8.19E-11 | structural constituent of cuticle |
| [ACG][AG]CTTA[GT]A. | 3.35E-06 | ion transport |
| [ACG][AG]CTTA[GT]A. | 1.22E-05 | structural molecule activity |
| [ACG][AG]CTTA[GT]A. | 2.25E-03 | cytoplasm |
| [ACT]CAC[AT]C[AC][CT]A | 1.75E-06 | ion channel activity |
| [ACT]CAC[AT]C[AC][CT]A | 1.75E-06 | alpha-type channel activity |
| [ACT]CAC[AT]C[AC][CT]A | 1.75E-06 | channel or pore class transporter activity |
| [ACT]CAC[AT]C[AC][CT]A | 5.20E-05 | ion transporter activity |
| [ACT]CAC[AT]C[AC][CT]A | 1.28E-04 | transporter activity |
| [ACT]CAC[AT]C[AC][CT]A | 4.22E-04 | signal transducer activity |
| [ACT]CAC[AT]C[AC][CT]A | 1.45E-03 | extracellular ligand-gated ion channel activity |
| [ACT]CAC[AT]C[AC][CT]A | 1.69E-03 | ligand-gated ion channel activity |
| [ACT]CAC[AT]C[AC][CT]A | 2.16E-03 | cation channel activity |
| [ACT]CAC[AT]C[AC][CT]A | 5.30E-03 | neurotransmitter binding |
| [ACT]CAC[AT]C[AC][CT]A | 5.30E-03 | neurotransmitter receptor activity |
| [ACT]CAC[AT]C[AC][CT]A | 9.65E-03 | synapse |
| [ACT]CAC[AT]C[AC][CT]A | 9.65E-03 | synapse part |
| [ACT]CAC[AT]C[AC][CT]A | 9.65E-03 | postsynaptic membrane |

| **Motifs identified in the 3’ UTR of the complete dataset** | | |
| --- | --- | --- |
| .[ACT][GT].CCCC. | 2.41E-06 | nucleotide binding |
| .[ACT][GT].CCCC. | 1.98E-04 | purine nucleotide binding |
| .[ACT][GT].CCCC. | 2.68E-03 | ATP binding |
| .[ACT][GT].CCCC. | 3.70E-03 | adenyl nucleotide binding |
| .A[AG][CT]AA[AG][CG]. | 1.75E-05 | sugar binding |
| .A[AG][CT]AA[AG][CG]. | 2.03E-05 | membrane part |
| .A[AG][CT]AA[AG][CG]. | 4.40E-05 | integral to membrane |
| .A[AG][CT]AA[AG][CG]. | 4.40E-05 | intrinsic to membrane |
| .A[AG][CT]AA[AG][CG]. | 9.85E-05 | carbohydrate binding |
| .A[AG][CT]AA[AG][CG]. | 8.59E-04 | cytoplasmic part |
| .A[AG][CT]AA[AG][CG]. | 4.28E-03 | transporter activity |
| .A[AG][CT]AA[AG][CG]. | 6.03E-03 | cytoplasm |
| .A[AG][CT]AA[AG][CG]. | 6.19E-03 | ribosome |
| .A[AG][CT]AA[AG][CG]. | 6.19E-03 | structural constituent of ribosome |
| .A[CG][ACG]TA[CGT][AG][ACT] | 2.90E-05 | signal transducer activity |
| .A[CG][ACG]TA[CGT][AG][ACT] | 7.24E-05 | receptor activity |
| .A[CG][ACG]TA[CGT][AG][ACT] | 6.35E-04 | integral to membrane |
| .A[CG][ACG]TA[CGT][AG][ACT] | 6.35E-04 | intrinsic to membrane |
| .A[CG][ACG]TA[CGT][AG][ACT] | 1.56E-03 | membrane part |
| .AA[AC]A[AC][CG]T[AGT] | 2.20E-04 | integral to membrane |
| .AA[AC]A[AC][CG]T[AGT] | 2.20E-04 | intrinsic to membrane |
| .AA[AC]A[AC][CG]T[AGT] | 6.76E-04 | transporter activity |
| .AA[AC]A[AC][CG]T[AGT] | 3.27E-03 | membrane part |
| [AG]A[CT]A[AG]AT[CT]. | 1.28E-05 | ion transport |
| [AG]A[CT]A[AG]AT[CT]. | 3.07E-05 | phosphate transport |
| [AG]A[CT]A[AG]AT[CT]. | 8.71E-05 | inorganic anion transport |
| [AG]A[CT]A[AG]AT[CT]. | 1.21E-04 | anion transport |
| [AG]A[CT]A[AG]AT[CT]. | 4.36E-03 | structural constituent of cuticle |
| .T[CT][CG][AT][CT]GT[GT] | 1.40E-08 | positive regulation of growth rate |
| .T[CT][CG][AT][CT]GT[GT] | 1.40E-08 | regulation of growth rate |
| .T[CT][CG][AT][CT]GT[GT] | 1.85E-08 | regulation of growth |
| .T[CT][CG][AT][CT]GT[GT] | 3.46E-08 | positive regulation of biological process |
| .T[CT][CG][AT][CT]GT[GT] | 5.90E-08 | positive regulation of growth |
| .T[CT][CG][AT][CT]GT[GT] | 2.59E-04 | reproduction |
| .T[CT][CG][AT][CT]GT[GT] | 4.62E-04 | gametogenesis |
| .T[CT][CG][AT][CT]GT[GT] | 8.45E-04 | sexual reproduction |
| .T[CT][CG][AT][CT]GT[GT] | 1.08E-03 | helicase activity |
| .T[CT][CG][AT][CT]GT[GT] | 2.95E-03 | genitalia development |
| .T[CT][CG][AT][CT]GT[GT] | 2.95E-03 | hermaphrodite genitalia development |
| .T[CT][CG][AT][CT]GT[GT] | 4.16E-03 | sex differentiation |
| .T[CT][CG][AT][CT]GT[GT] | 5.20E-03 | organ development |
| .T[CT][CG][AT][CT]GT[GT] | 5.27E-03 | ATP-dependent helicase activity |
| [GT][GT]TA[AT][AC][GT][AC][GT] | 3.49E-03 | helicase activity |
| .A[AC]ATAA[CT][AGT] | 1.33E-11 | phosphate transport |
| .A[AC]ATAA[CT][AGT] | 3.01E-11 | inorganic anion transport |
| .A[AC]ATAA[CT][AGT] | 3.91E-11 | anion transport |
| .A[AC]ATAA[CT][AGT] | 4.45E-07 | structural constituent of cuticle |
| .A[AC]ATAA[CT][AGT] | 2.03E-05 | ion transport |
| .A[AC]ATAA[CT][AGT] | 1.64E-03 | cytoplasm |
| .A[AC]ATAA[CT][AGT] | 4.76E-03 | structural molecule activity |

| **Motifs identified in the 5’ end of the egg-L4 dataset** | | |
| --- | --- | --- |
| [CGT]CG[AC]G[AGT]C[CG][CGT] | 1.07E-07 | growth |
| [CGT]CG[AC]G[AGT]C[CG][CGT] | 5.45E-07 | development |
| [CGT]CG[AC]G[AGT]C[CG][CGT] | 1.28E-06 | reproduction |
| [CGT]CG[AC]G[AGT]C[CG][CGT] | 2.91E-06 | embryonic development |
| [CGT]CG[AC]G[AGT]C[CG][CGT] | 2.91E-06 | embryonic development (sensu Metazoa) |
| [CGT]CG[AC]G[AGT]C[CG][CGT] | 3.18E-06 | positive regulation of growth |
| [CGT]CG[AC]G[AGT]C[CG][CGT] | 4.25E-06 | regulation of growth |
| [CGT]CG[AC]G[AGT]C[CG][CGT] | 4.25E-06 | positive regulation of biological process |
| [CGT]CG[AC]G[AGT]C[CG][CGT] | 4.66E-06 | positive regulation of growth rate |
| [CGT]CG[AC]G[AGT]C[CG][CGT] | 4.66E-06 | regulation of growth rate |
| [CGT]CG[AC]G[AGT]C[CG][CGT] | 3.59E-05 | nucleotide binding |
| [CGT]CG[AC]G[AGT]C[CG][CGT] | 7.72E-05 | nucleoside-triphosphatase activity |
| [CGT]CG[AC]G[AGT]C[CG][CGT] | 1.91E-04 | pyrophosphatase activity |
| [CGT]CG[AC]G[AGT]C[CG][CGT] | 1.91E-04 | hydrolase activity, acting on acid anhydrides |
| [CGT]CG[AC]G[AGT]C[CG][CGT] | 1.91E-04 | hydrolase activity, acting on acid anhydrides |
| [CGT]CG[AC]G[AGT]C[CG][CGT] | 2.48E-04 | nucleic acid binding |
| [CGT]CG[AC]G[AGT]C[CG][CGT] | 3.11E-04 | purine nucleotide binding |
| [CGT]CG[AC]G[AGT]C[CG][CGT] | 9.08E-04 | regulation of biological process |
| [CGT]CG[AC]G[AGT]C[CG][CGT] | 1.03E-03 | adenyl nucleotide binding |
| [CGT]CG[AC]G[AGT]C[CG][CGT] | 1.62E-03 | ATP binding |
| [CGT]CG[AC]G[AGT]C[CG][CGT] | 2.64E-03 | RNA binding |
| [CGT]CG[AC]G[AGT]C[CG][CGT] | 6.89E-03 | response to stimulus |
| [CGT]CG[AC]G[AGT]C[CG][CGT] | 7.34E-03 | organ development |
| [CGT]CG[AC]G[AGT]C[CG][CGT] | 8.52E-03 | gametogenesis |
| .[AC][ACT]CGCTC. | 1.71E-05 | embryonic development |
| .[AC][ACT]CGCTC. | 1.71E-05 | embryonic development (sensu Metazoa) |
| .[AC][ACT]CGCTC. | 2.76E-04 | development |
| .TTC[CG]C[AG]C[ACG] | 5.97E-04 | development |
| .TTC[CG]C[AG]C[ACG] | 5.98E-04 | embryonic development |
| .TTC[CG]C[AG]C[ACG] | 5.98E-04 | embryonic development (sensu Metazoa) |
| .CTGAAAA. | 2.38E-07 | development |
| .CTGAAAA. | 2.84E-06 | embryonic development |
| .CTGAAAA. | 2.84E-06 | embryonic development (sensu Metazoa) |
| [ACT][ACG]CGTGA[AC]. | 1.02E-06 | phosphate metabolism |
| [ACT][ACG]CGTGA[AC]. | 1.02E-06 | phosphorus metabolism |
| [ACT][ACG]CGTGA[AC]. | 1.15E-04 | phosphorylation |
| [ACT][ACG]CGTGA[AC]. | 2.53E-04 | protein amino acid phosphorylation |
| [ACT][ACG]CGTGA[AC]. | 4.72E-04 | protein kinase activity |
| [ACT][ACG]CGTGA[AC]. | 1.08E-03 | kinase activity |
| [ACT][ACG]CGTGA[AC]. | 1.90E-03 | transferase activity, phosphorus-containing groups |
| [ACT][ACG]CGTGA[AC]. | 4.00E-03 | phosphotransferase activity, alcohol group as acceptor |
| .ATA[CG]ATA[CGT] | 5.73E-03 | structural constituent of cuticle |
| .ATCAAAA. | 3.21E-05 | phosphate transport |
| .ATCAAAA. | 5.27E-05 | inorganic anion transport |
| .ATCAAAA. | 2.05E-04 | anion transport |
| .ATCAAAA. | 7.59E-04 | structural constituent of cuticle |
| .AACTTTG. | 3.16E-07 | integral to membrane |
| .AACTTTG. | 3.16E-07 | intrinsic to membrane |
| .AACTTTG. | 3.55E-07 | membrane part |
| .AACTTTG. | 4.10E-07 | membrane |
| .AACTTTG. | 1.01E-05 | signal transducer activity |
| .AACTTTG. | 1.21E-05 | receptor activity |
| .AACTTTG. | 7.73E-05 | G-protein coupled receptor protein signaling pathway |
| .AACTTTG. | 3.12E-04 | cell surface receptor linked signal transduction |
| .AACTTTG. | 3.81E-04 | rhodopsin-like receptor activity |
| .AACTTTG. | 5.86E-04 | transmembrane receptor activity |
| .AACTTTG. | 2.53E-03 | G-protein coupled receptor activity |
| .AACTTTG. | 4.82E-03 | transporter activity |
| .AACTTTG. | 6.96E-03 | ion channel activity |
| .AACTTTG. | 6.96E-03 | alpha-type channel activity |
| .AACTTTG. | 6.96E-03 | channel or pore class transporter activity |

| **Motifs from the 3’UTR of the egg-L4 dataset** | | |
| --- | --- | --- |
| ..[AT][CT]CCCC. | 7.81E-04 | development |
| ..[AT][CT]CCCC. | 1.70E-03 | growth |
| ..[AT][CT]CCCC. | 9.96E-03 | regulation of biological process |
| .A[AC]TAA[AG]T. | 2.83E-08 | structural molecule activity |
| .A[AC]TAA[AG]T. | 9.94E-03 | structural constituent of cuticle |
| .GA[AC][CT][AGT]G[AC][ACG] | 4.85E-04 | protein tyrosine phosphatase activity |
| .GA[AC][CT][AGT]G[AC][ACG] | 5.31E-04 | phosphate metabolism |
| .GA[AC][CT][AGT]G[AC][ACG] | 5.31E-04 | phosphorus metabolism |
| .GA[AC][CT][AGT]G[AC][ACG] | 1.38E-03 | protein modification |
| .GA[AC][CT][AGT]G[AC][ACG] | 1.76E-03 | biopolymer modification |
| .GA[AC][CT][AGT]G[AC][ACG] | 1.94E-03 | phosphoprotein phosphatase activity |
| .GA[AC][CT][AGT]G[AC][ACG] | 1.94E-03 | protein amino acid dephosphorylation |
| .GA[AC][CT][AGT]G[AC][ACG] | 1.94E-03 | dephosphorylation |
| .GA[AC][CT][AGT]G[AC][ACG] | 8.31E-03 | phosphoric monoester hydrolase activity |
| .[ACT]GA[AG]G[ACT][GT][ACG] | 4.53E-07 | phosphate metabolism |
| .[ACT]GA[AG]G[ACT][GT][ACG] | 4.53E-07 | phosphorus metabolism |
| .[ACT]GA[AG]G[ACT][GT][ACG] | 1.07E-04 | protein modification |
| .[ACT]GA[AG]G[ACT][GT][ACG] | 1.48E-04 | biopolymer modification |
| .[ACT]GA[AG]G[ACT][GT][ACG] | 7.19E-04 | biopolymer metabolism |
| .[ACT]GA[AG]G[ACT][GT][ACG] | 1.44E-03 | protein amino acid phosphorylation |
| .[ACT]GA[AG]G[ACT][GT][ACG] | 1.85E-03 | protein kinase activity |
| .[ACT]GA[AG]G[ACT][GT][ACG] | 1.89E-03 | phosphotransferase activity, alcohol group as acceptor |
| .[ACT]GA[AG]G[ACT][GT][ACG] | 2.93E-03 | kinase activity |
| .[ACT]GA[AG]G[ACT][GT][ACG] | 3.82E-03 | phosphorylation |
| .[ACT]GA[AG]G[ACT][GT][ACG] | 5.03E-03 | protein tyrosine phosphatase activity |
| .[ACT]GA[AG]G[ACT][GT][ACG] | 7.56E-03 | structural molecule activity |
| .A[AG][AT]AC[AC]A. | 8.21E-05 | signal transducer activity |
| .A[AG][AT]AC[AC]A. | 1.85E-04 | receptor activity |
| .A[AG][AT]AC[AC]A. | 7.24E-04 | integral to membrane |
| .A[AG][AT]AC[AC]A. | 7.24E-04 | intrinsic to membrane |
| .A[AG][AT]AC[AC]A. | 8.25E-04 | membrane |
| .A[AG][AT]AC[AC]A. | 1.43E-03 | neurotransmitter binding |
| .A[AG][AT]AC[AC]A. | 1.43E-03 | neurotransmitter receptor activity |
| .A[AG][AT]AC[AC]A. | 3.54E-03 | membrane part |
| .[AC]T[AG][AG][AG]CA. | 6.91E-06 | phosphate transport |
| .[AC]T[AG][AG][AG]CA. | 1.00E-05 | inorganic anion transport |
| .[AC]T[AG][AG][AG]CA. | 2.83E-05 | anion transport |
| .[AC]T[AG][AG][AG]CA. | 1.48E-03 | structural constituent of cuticle |
